# A phase Ib dose-escalation study of troriluzole (BHV-4157), an oral glutamatergic signaling modulator, in combination with nivolumab in patients with advanced solid tumors

**DOI:** 10.1186/s40001-022-00732-w

**Published:** 2022-07-02

**Authors:** Ann W. Silk, Biren Saraiya, Roman Groisberg, Nancy Chan, Kristen Spencer, Eugenia Girda, Weichung Shih, Marisa Palmeri, Tracie Saunders, Robert M. Berman, Vlad Coric, Suzie Chen, Andrew Zloza, Joshua Vieth, Janice M. Mehnert, Jyoti Malhotra

**Affiliations:** 1 Dana-Farber Cancer Institute and Harvard Medical School, 450 Brookline Ave, Room LW503, Boston, MA USA; 2 Rutgers Cancer Institute of New Jersey and Robert Wood Johnson Medical School, New Brunswick, NJ USA; 3 Laura and Isaac Perlmutter Cancer Center and New York University Grossman School of Medicine, New York, NY USA; 4 Rutgers University School of Public Health, New Brunswick, NJ USA; 5 Chi-Square Consulting LLC, Piscataway, NJ USA; 6 Biohaven Pharmaceuticals, New Haven, CT USA; 7 Rutgers University School of Pharmacy, Piscataway, NJ USA; 8 Rush University Medical Center and Department of Internal Medicine, Rush Medical College, Chicago, IL USA; 9 JDRF International, New York, NY USA

**Keywords:** Glutamate, Prodrug, Immunotherapy resistance

## Abstract

**Background:**

Glutamate signaling activates MAPK and PI3K/AKT pathways in tumor cells. Treatment with riluzole, a glutamate release inhibitor, has been previously shown to be safe in melanoma patients and produced biologic effects, but did not lead to radiographic responses, possibly due to poor pharmacokinetic properties. Therefore, we conducted a phase Ib trial to determine the safety and tolerability of the combination of the riluzole prodrug troriluzole (BHV-4157, trigriluzole) and the PD-1 antibody nivolumab in patients with advanced solid tumors.

**Methods:**

Patients with advanced or refractory solid tumors and measurable disease per RECIST 1.1 were treated with increasing doses of troriluzole using a semi-Bayesian modified toxicity probability interval dose escalation procedure. Troriluzole monotherapy was orally self-administered for a 14-day lead-in period followed by continuation of troriluzole in combination with nivolumab 240 mg IV every 2 weeks. Endpoints included safety, pharmacokinetics (PK) and efficacy.

**Results:**

We enrolled 14 patients with advanced solid tumors (melanoma = 3, NSCLC = 3, renal cell carcinoma = 2, bladder/urothelial = 2, ovarian cancer = 1, adenoid cystic carcinoma = 1, pleural mesothelial = 1, head and neck cancer = 1). Eleven patients had cancer progression on prior therapy with PD-1 or PD-L1 agent. Patients received troriluzole total daily doses from 140 to 560 mg (divided). The most common treatment-related adverse events (TRAE) occurring in ≥ 5 patients (> 35%) were transaminitis and increased lipase. DLT (dose-limiting toxicity) occurred in 3 patients: (1) grade 3 anorexia, (2) grade 3 fatigue and, (3) grade 3 atrial fibrillation. Six patients were treated at the MTD (maximum tolerated dose). No subjects discontinued treatment due to AEs. One response occurred (7%), which was a partial response in a subject who had PD-1 refractory disease. The 6-month PFS rate was 21%. PK data showed that the prodrug troriluzole was efficiently cleaved into riluzole by 2-h post-dosing in all dose cohorts tested.

**Conclusion:**

The combination of troriluzole and nivolumab was safe and well-tolerated. The MTD of troriluzole was determined to be 420 mg total daily dose. The observed antitumor activity, primarily disease stabilization, is of interest in patients with PD-1 resistant tumors.

*Trial Registration* ClinicalTrials.gov Identifier NCT03229278.

## Introduction

The glutamate signaling pathway promotes tumor growth, angiogenesis, migration, and invasiveness in a variety of cancers, including melanoma [1, 2], colon adenocarcinoma [3], breast cancer [4], gliomas [5–8], and non-small cell lung cancer [9]. Glutamate activates the metabotropic glutamate receptor family (mGluR), which is found on normal neurons as well as cancer cells. In tumors, activation of mGluR1, a member of mGluR family, results in stimulation of the PKC, MAPK, and PI3K/AKT pathways [1, 2].

Riluzole is a glutamatergic signaling-modulating drug that was FDA-approved in 1995 for the treatment of amyotrophic lateral sclerosis (ALS) [10]. One of the actions of riluzole is to block the release of glutamate from cells, thus limiting extracellular levels of glutamate, thereby acting as a functional inhibitor of mGluR1 [1]. Through a mechanism that is not entirely understood, riluzole also modulates the sodium-dependent currents in mammalian neurons in a dose-dependent manner [11].

Recently, there has been interest in re-purposing riluzole as a cancer therapy. Effects in humans were investigated in a phase 0 trial in which patients with advanced melanoma were treated in a neoadjuvant manner with riluzole 100 mg orally twice a day for 2 weeks while awaiting surgery [12]. Four out of 12 patients (34%) exhibited decreased FDG-avidity on PET scans despite a short duration of treatment, and the same patients also demonstrated a decrease in phosphorylated AKT and/or ERK in paired pre-/post-treatment tissue samples. In a phase II trial of riluzole of 13 patients with metastatic melanoma, no objective responses were observed; however, 6/13 patients who entered the trial with rapidly progressive disease achieved stable disease, and 4 of those 6 patients maintained clinical benefit of 6–13 months without progression of disease [13]. In a subset of 4 patients with sufficient paired pre-/post-treatment tissue for IHC analysis, 2 samples from patients with stable disease displayed an increase in CD45 + leukocytes at the tumor–stromal interface, as well as post-treatment decreases in phosphorylated ERK, phosphorylated AKT, and CD31 by Western blot analysis. The other two patients had progressive disease and did not demonstrate any of these pharmacodynamic effects. The patterns of these exploratory correlative studies suggests that MAPK and PI3K/AKT down-regulation and increased leukocytes at the active edge of tumor correlated with clinical benefit. This observation is clinically significant because the presence of tumor infiltrating lymphocytes in the microenvironment of the tumor is associated with increased efficacy of immune checkpoint inhibitors [14–16].

We hypothesized that inhibiting glutamate signal transduction could prime the tumor microenvironment to respond more favorably to anti-PD-1 immune checkpoint antibodies. Preclinical data support this mechanism. Using an immunocompetent GRM-1 allograft melanoma mouse model [2], riluzole inhibited tumor growth, and growth was further inhibited in combination with PD-1 blockade [17].

Riluzole has poor bioavailability (60%) and the concentration is affected by food and variability of CYP1A2 expression [18]. Troriluzole is a third-generation prodrug of riluzole that bypasses the liver and is rapidly cleaved by circulating blood plasma to yield the active compound riluzole. Troriluzole has good bioavailability and no food effect. In a mouse model of glioblastoma with GL261 glioma cells implanted intracranially, treatment with troriluzole significantly improved survival compared to the control arm, and combined treatment with troriluzole and an anti-PD-1 antibody significantly improved survival compared to the control arm [19]. Sampling of the tumor microenvironment demonstrated an increase in CD4 + T cells and a decrease in Foxp3 + T cells in mice treated with troriluzole, and depletion studies confirmed an immune-mediated mechanism. Based on this work, we conducted a Phase Ib study of troriluzole in combination with the anti-PD-1 agent nivolumab in patients with advanced solid tumors (NCT03229278).

## Methods

### Study population

Patients were enrolled in this trial between October 2017 and February 2019. Patients were eligible if they were 18 years of age or older with refractory solid tumor for which nivolumab is indicated. Patients were required to complete all prior chemotherapy, immunotherapy, radiotherapy, or major surgery at least 3 weeks before treatment start. Other eligibility criteria included an ECOG performance status of 0–2, measurable disease per RECIST version 1.1, and adequate organ and marrow function. Prior treatment with anti-PD-(L) 1 agent was allowed. Exclusion criteria included systemic immunosuppressive medications, ongoing immune-related adverse event from prior immunotherapy that did not improve to grade 1 or better (with exceptions for grade 2 hypothyroidism and adrenal insufficiency), and serious concomitant disorders such as active infection. Additional exclusion criteria included corticosteroid treatment of greater than 10 mg daily within 14 days of treatment start, HIV diagnosis, second active primary malignancy, active and untreated brain metastases, pregnancy, or use of medications that are CYP1A2 inhibitors (including cimetidine, amiodarone, and fluoroquinolones).

### Study design

This was a single-institution Phase I sequential dose-escalation study. Patients were assigned to dose-escalation cohorts in increasing doses of troriluzole, which was self-administered, with or without food. The starting dose of troriluzole for the first cohort was 140 mg daily. This dose was selected because it is the molar equivalent of 70 mg of riluzole, which is less than the FDA-approved dose of riluzole (100 mg daily). In the absence of dose-limiting toxicity (DLT), dose escalation was designed to proceed to a pre-specified maximum dose of 280 mg of troriluzole twice a day (Table 1). The primary endpoint of the trial was to assess safety and tolerability of the treatment regimen and to determine the maximum tolerable dose/recommended phase 2 dose (MTD/RP2D). Secondary endpoints of the trial were overall response rate (ORR) and progression-free survival response (PFS).

**Table 1:** Dose-escalation cohorts and DLTs

Dosing cohort	Troriluzole, continuous oral dosing	Nivolumab q 2 weeks	Patients treated (*n*)	DLTs (*n*)
Cohort 1	140 mg PO QHS	240 mg	3	0
Cohort 2	140 mg PO QAM 140 mg PO QHS	240 mg	6	1 (anorexia)
Cohort 3	140 mg PO QAM 280 mg PO QHS	240 mg	3	0
Cohort 4	280 mg PO QAM 280 mg PO QHS	240 mg	2	2 (fatigue and atrial fibrillation)

### Study treatment

**Fig. 1:**
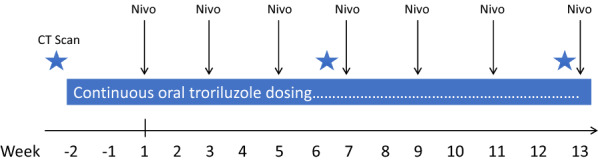
Study schema. After registration, participants had a lead-in period of troriluzole monotherapy for 2 weeks to assess tolerance. Nivolumab was added at Week 1 and infusions were every 2 weeks in combination with troriluzole. Tumor assessments were performed by CT scan at baseline, Week 6, Week 12 and every 12 weeks for a year (not shown, schema is truncated for clarity)

Troriluzole monotherapy was given for a 14-day lead-in period (Weeks 2 and 1) to assess for toxicity. After completion of the lead-in (Week 1), nivolumab at the standard dose of 240 mg IV every 2 weeks was administered in combination (Fig. 1). Toxicities were graded by the Common Terminology Criteria for Adverse Events (CTCAE) version 4. Grade 3 to 4 toxicities attributed to troriluzole required holding the medication and resuming it with a dose reduction of one level. There were no dose reductions allowed for nivolumab. If a grade 3 or 4 immune-related adverse event occurred, then nivolumab was held until the toxicity was grade 1 or less, and a corticosteroid with a maximum 8-week taper was initiated. Exceptions included grade 3 or higher elevated amylase or lipase for which there were no clinical symptoms.

### Response assessment

Restaging scans were performed at Week 7, Week 13, and every 12 weeks thereafter. Radiologic assessments were evaluated using RECIST 1.1. All responses had to be confirmed 4 to 12 weeks later, but progressive disease was confirmed at the first instance if the patient exhibited clinical deterioration (decrease in performance status).

### Correlative analyses

Blood samples were collected for PK analyses on the first day of treatment at three timepoints: at pre-treatment and at 2 and 4 h post troriluzole administration at Week 2 (first day of troriluzole treatment), Week 1 (completion of lead-in period) and Week 7. Samples were stored at less than – 20 °C for up to 30 days and shipped to inVentiv Health (Québec, QC, Canada) for analysis. Additional correlative samples were collected at the same timepoints for cytokine and other analysis, which were processed and stored by the Rutgers Cancer Institute of New Jersey (RCINJ) Biospecimen Repository and Histology Service shared resource. The immune assays were performed at the RCINJ Immune Monitoring and Advanced Genomics Shared Resource Services. Luminex cytokine analysis was performed on paired serum samples with a 48-plex human Cytokine/Chemokine/Growth Factor Panel A (Millipore Sigma, Burlington, MA) analyzed on a Luminex 2000 analyzer (Luminex Corporation, Austin, TX).

### Statistical analysis

The dose escalation proceeded according to the semi-Bayesian modified toxicity probability interval (mTPI) method [20]. The starting cohort size was 3 patients. The occurrence of any Grade 3 or higher toxicity during the DLT evaluation period (the first 5 weeks) was considered a DLT, if judged by the treating investigator to be related to the administration of either study drug or the combination of study drugs. There was no within-patient dose escalation allowed. AEs were described using a frequency table. PK data were plotted for each patient. Subjects were divided in half by PFS (< 4 months vs.  ≥ 4 months) for analysis of baseline and Week 7 circulating cytokines, and levels were compared using the Welch’s *T* test.

## Results

Fourteen patients received treatment as per dose cohorts in Table 2 (melanoma = 3, NSCLC = 3, renal cell carcinoma = 2, bladder/urothelial = 2, ovarian cancer = 1, adenoid cystic carcinoma = 1, pleural mesothelial = 1, head and neck cancer = 1). Males accounted for 57% of the study population and median age at enrollment was 65 years. Eleven (79%) patients had cancer that had progressed on therapy with an anti-PD-1 or PD-L1 agent prior to study entry.

**Table 2:** Baseline characteristics

	*n*	%
Gender		
Male	8	57.1
Female	6	42.9
Race/ethnicity		
Non-Hispanic White	9	64.3
African American	2	14.3
Hispanic	3	21.4
Age, years		
Median (SD)	65.0 (9.3)	
Range	52—81	
ECOG performance status		
0	4	28.6
1	10	71.4
Primary tumor		
Melanoma	3	21
NSCLC	3	21
Renal	2	14
Bladder/urothelial	2	14
Ovary	1	7
Pleural mesothelioma	1	7
Adenoid cystic	1	7
Head and neck	1	7

Median treatment duration was 12 weeks (range 6 to 48 weeks). The most frequent TRAE (all grades) were elevations in ALT (42.9%), AST (35.7%), and lipase (35.7%; Table 3). A total of 4 high-grade TRAE occurred, which were elevated AST (grade 3), elevated lipase (grades 3 and 4), and fatigue (grade 3). No patient required more than one dose reduction. There were no treatment-related therapy discontinuations or treatment-related deaths. Three patients had DLTs (Table 2), including grade 3 anorexia (cohort 2; 280 mg total daily dose), grade 3 fatigue and grade 3 atrial fibrillation (both subjects in cohort 4; 560 mg total daily dose). Because 2/2 patients enrolled in cohort 4 experienced a DLT, cohort 3 (420 mg total daily dose) was determined to be MTD.

**Table 3:** Treatment-related adverse events experienced by ≥ 20% patients

Adverse event	All grades *n* (%)	Gr 2, *n* (%)	Gr 3, *n* (%)	Gr 4, *n* (%)
Elevated ALT	6 (42.9)	1 (7.1)	0 (0.0)	0 (0.0)
Elevated AST	5 (35.7)	1 (7.1)	1 (7.1)	0 (0.0)
Lipase increased	5 (35.7)	2 (14.3)	1 (7.1)	1 (7.1)
Fatigue	4 (28.6)	2 (14.2)	1 (7.1)	0 (0.0)
Diarrhea	3 (21.4)	1 (7.1)	0 (0.0)	0 (0.0)
Lymphocyte count decreased	3 (21.4)	2 (14.3)	0 (0.0)	0 (0.0)
Nausea	3 (21.4)	3 (21.4)	0 (0.0)	0 (0.0)
Platelet count decreased	3 (21.4)	0 (0.0)	0 (0.0)	0 (0.0)

One patient (7%), who had bladder carcinoma had had received prior anti-PD-1 therapy, in cohort 3 experienced a confirmed partial response (PR)*.* Additionally, 3 patients (21%) had stable disease lasting > 6 months, including a PD-1 refractory RCC patient who had SD duration of 48 weeks*.* Durable clinical benefit rate was 29% (PR or SD lasting more than 6 months).

**Fig. 2:**
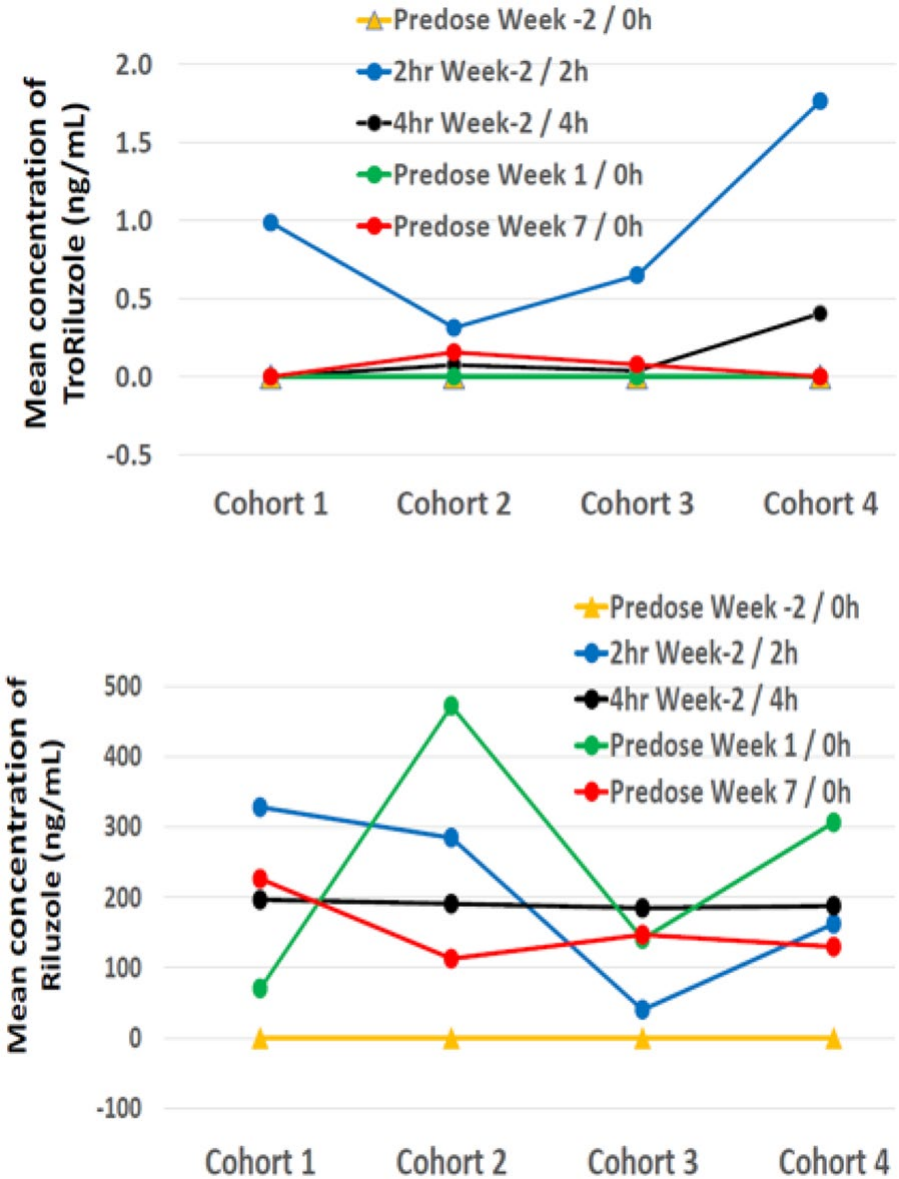
Troriluzole and riluzole concentrations (ng/mL) in human EDTA K2 plasma, average per dosing cohort taken before and 2 and 4 h after oral dosing at Week 2 (the beginning of troriluzole treatment), and pre-dose at Week 1 (before nivolumab) and Week 7 (after treatment with nivolumab)

PK analysis demonstrated that little to no troriluzole was detected 2–4 h after administration, as expected (Fig. 2A). Plasma concentrations of the active compound riluzole were detectable at the 2-h and 4-h post-dose timepoints, and under steady-state conditions (Week 1 and 7 on-study pre-dose troughs; Fig. 2B). Mean concentrations of riluzole were greater than 100 ng/mL in most dosing cohorts. No clear increase in mean concentration with increasing dose was observed.

**Fig. 3:**
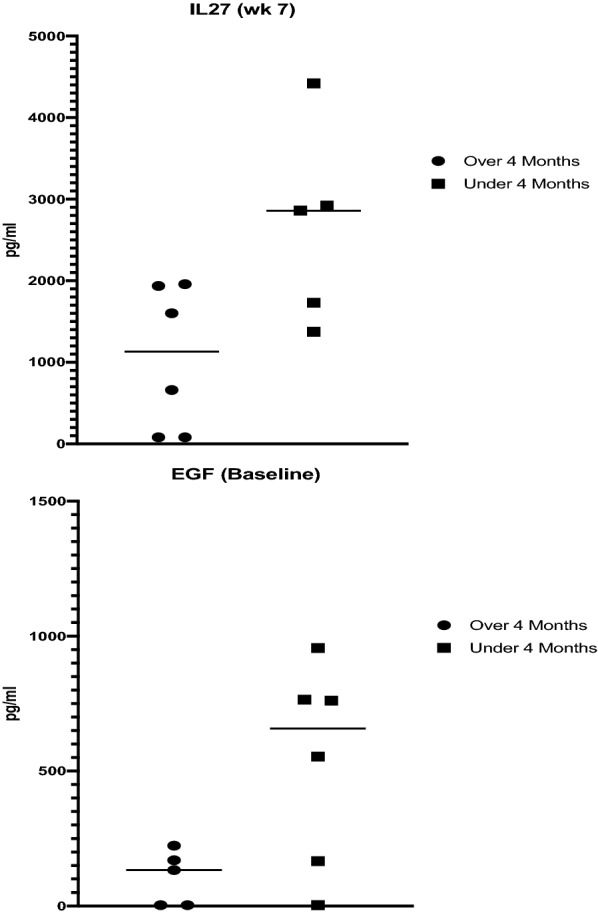
Multiplex cytokine analysis in all available samples from patients with PFS of≥ 4 months vs. < 4 months. At baseline, patients with PFS ≥ 4 months had significantly lower levels of epidermal growth factor (EGF; *p* = 0.0372; Welch’s *T* test; **A**). Patients with PFS ≥ 4 months had significantly lower levels of interleukin-27 (IL-27) at week 7 (*p* = 0.0403, Welch’s *T* test; **B**)

Multiplex cytokine analysis demonstrated that patients with PFS of ≥ 4 months had significantly lower levels of epidermal growth factor (EGF) at baseline (*p* = 0.0372; Welch’s T test; Fig. 3A). Patients with PFS ≥ 4 months had significantly lower levels of interleukin-27 (IL-27) at week 7 (*p* = 0.0403, Welch’s *T* test; Fig. 3B). There were no other significant associations in the other 45 cytokine levels in the panel. Unfortunately, PBMC immunophenotyping could not be performed because the samples failed quality analysis due to improper processing and storage.

## Discussion

The combination of troriluzole and nivolumab was safe and well-tolerated. The MTD was determined to be troriluzole 420 mg PO total daily dose (troriluzole 140 mg QAM + 280 mg PO QHS) in combination with standard dose nivolumab. Spot PK sampling demonstrated that the prodrug was efficiently cleaved, regardless of food intake, to yield circulating levels of riluzole with mean concentrations in the range of 100–200 ng/mL. There was no clear association between mean concentration and increasing dose, possibly because maximum concentration was not captured due to the limited PK collection schedule.

Modest clinical benefit was observed. Six (43%) of the 14 subjects had disease control for at least 4 months, one of whom was a nivolumab-refractory renal cell carcinoma subject who experienced disease stabilization for more than 11 months. We observed one objective partial response in a patient with bladder carcinoma cancer previously treated with the anti-PD-L1 antibody atezolizumab. Unfortunately, this subject was lost to follow-up at 4 months because he relocated.

Cytokines were assayed before and during treatment. Low EGF in baseline blood samples was associated with improved PFS (*p* = 0.0372). In laboratory experiments using ovarian cancer and head and neck cancer cell lines, EGF promoted TAMS and metastasis [21, 22]. Based on our data, we cannot conclude if low pre-treatment EGF level is predictive or prognostic, but EGF levels may be worthy of further investigation. During treatment at Week 7, there was a significant association between low levels of IL-27 and improved PFS (*p* = 0.0403). IL-27 is a member of the IL-12 cytokine family and many of its biologic effects overlap with those of interferon-γ. However, IL-27 also promotes tumor survival and growth through induction of regulatory T cells and modulation of tumor-associated macrophages through up-regulation of CD39 or PD-L1 [23, 24]. Low IL-27 levels in subjects who benefitted from troriluzole and nivolumab may reflect the dual role of IL-27 in cancer immunotherapy.

## Conclusions

In summary, treatment with troriluzole plus a PD-1 inhibitor demonstrated a favorable safety profile in combination with nivolumab, and treatment resulted in expected concentrations of the active metabolite riluzole in a population of subjects with refractory cancers. The MTD of troriluzole was 420 mg, which is six times the molar equivalent of the usual dose of riluzole for ALS. The observed antitumor activity, primarily disease stabilization, is of interest in patients with PD-1 resistant tumors. The combination will be further investigated in a randomized phase II trial in metastatic melanoma with brain metastases (NCT04899921).

## Data Availability

The data that support the findings of this study are available upon reasonable request from Ms. Tracie Saunders.

## Abbreviations

ALS: Amyotrophic lateral sclerosis
GRM-1: Glutamate receptor metabotropic 1 gene
NSCLC: Non-small cell lung cancer
MTD: Maximum tolerated dose
mGluR: Metabotropic glutamate receptor family of proteins
mTPI: Modified toxicity probability interval
PK: Pharmacokinetic
PD-1: Programmed death-1
PD-L1: Programmed death-1 ligand
RECIST: Response evaluation criteria in solid tumors
SD: Standard deviation
TRAE: Treatment-related adverse events
